# Allergen Recognition by Innate Immune Cells: Critical Role of Dendritic and Epithelial Cells

**DOI:** 10.3389/fimmu.2013.00356

**Published:** 2013-11-04

**Authors:** Fabián Salazar, Amir M. Ghaemmaghami

**Affiliations:** ^1^Division of Immunology, Faculty of Medicine and Health Sciences, The University of Nottingham, Nottingham, UK

**Keywords:** dendritic cell, epithelial cell, asthma, allergy, type-I hypersensitivity, house dust mite, pattern recognition receptor, TSLP

## Abstract

Allergy is an exacerbated response of the immune system against non-self-proteins called allergens and is typically characterized by biased type-2 T helper cell and deleterious IgE mediated immune responses. The allergic cascade starts with the recognition of allergens by antigen presenting cells, mainly dendritic cells (DCs), leading to Th2 polarization, switching to IgE production by B cells, culminating in mast cell sensitization and triggering. DCs have been demonstrated to play a crucial role in orchestrating allergic diseases. Using different C-type lectin receptors DCs are able to recognize and internalize a number of allergens from diverse sources leading to sensitization. Furthermore, there is increasing evidence highlighting the role of epithelial cells in triggering and modulating immune responses to allergens. As well as providing a physical barrier, epithelial cells can interact with allergens and influence DCs behavior through the release of a number of Th2 promoting cytokines. In this review we will summarize current understanding of how allergens are recognized by DCs and epithelial cells and what are the consequences of such interaction in the context of allergic sensitization and downstream events leading to allergic inflammation. Better understanding of the molecular mechanisms of allergen recognition and associated signaling pathways could enable developing more effective therapeutic strategies that target the initial steps of allergic sensitization hence hindering development or progression of allergic diseases.

## Introduction

Asthma is a chronic disease of the lung characterized by inflammation and airway hyper-responsiveness. Allergic asthma is probably the most common form of asthma and is classified as a type-I hypersensitivity reaction. Most pathologies that are associated with allergic asthma are the consequence of an exacerbated immune response to specific proteins known as allergens in genetically susceptible individuals (1, 2). An allergic reaction is characterized by the synthesis of allergen-specific immunoglobulin of the IgE class and Th2 cytokines (e.g., IL-4, IL-5, and IL-13), which lead to recruitment and sensitization of effector cells such as eosinophils, basophils, and mast cells (1, 3). During allergen re-exposure, the crosslinking of IgE molecules bounded to high-affinity Fcϵ receptors (FcϵR) on the surface of mast cells and basophils results in an immediate release of the soluble mediators, such as histamine, leukotriene, and prostaglandins, which are responsible for the allergic reaction (1, 4).

Antigen recognition and uptake by innate immune cells is the first step in the process of antigen presentation that could lead to initiation of adaptive immune responses. Using a diverse set of pattern recognition receptors (PRRs) such as Toll-like receptors (TLRs) and C-type lectin receptors different types of immune and non-immune cells are able to sense conserved motifs on antigens. Dendritic cells (DCs) have been demonstrated to play a pivotal role in this process; however, the molecular mechanisms of how Th2-driven allergic immune responses are initiated and amplified have remained elusive (3–5). Recently, the role of epithelial cells as key modulators of DC behavior has been highlighted (6, 7). Specifically, airway epithelial cells (AECs) have been demonstrated to be able to recognize diverse allergens leading to the release of chemokines, cytokines, and danger signals that activate and recruit other immune cells to the site of inflammation (6, 7).

In this review, we will discuss the role of dendritic and epithelial cells in allergen recognition and how the cross-talk between DCs and AEC could affect Th2-mediated allergic diseases.

## Allergen Recognition by Dendritic Cells

**Figure 1:**
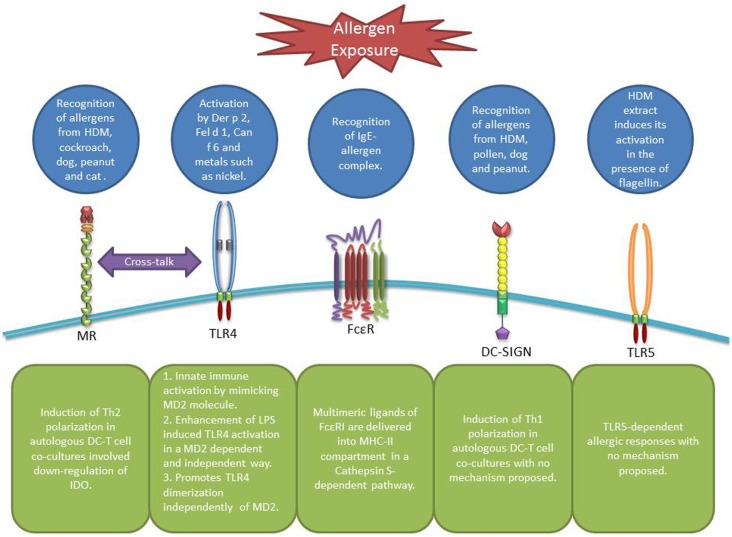
**Receptors involved in allergen recognition and uptake by DCs**. MR can recognize allergens from diverse sources leading to Th2 polarization and IDO down-regulation in DCs involving a possible cross-talk with TLR4 (19). TLR4 itself can be activated by airborne allergens and diverse metals in an MD2 dependent or independent way (33, 35–37). FcϵR facilitate allergen uptake through the recognition of the IgE-allergen complex (41, 46). DC-SIGN can recognize diverse allergens as well, however, leading to a Th1 polarization. Finally, TLR5 can be activated by HDM extracts containing flagellin (34).

Immature DCs reside in the peripheral tissues and can efficiently sample the microenvironment for antigens. Once taken up by DCs such antigens are processed into peptides and appear on the surface of DCs in the context of MHC molecules. Antigen bearing DC migrate to the local lymph nodes where through expression of MHCII-peptide complex, cytokines, and co-stimulatory molecules they can stimulate naïve T cells toward distinct effector T cell subsets (e.g., Th1, Th2, Th17) (8, 9) or induce tolerance through induction of regulatory T cells (10), depending on the nature of the antigen and other microenvironmental factors (11). DCs serve as sentinels of the mucosal surfaces, where they constantly sample antigens at the interface between external and internal environments using different PRRs (Figure 1). Even intraepithelial DCs are able to form tight junctions with epithelial cells through expression of proteins like occludin and claudin, which can further facilitate antigen/allergen recognition and uptake by these cells (7, 8). In addition, some allergens can gain access to DCs by disrupting the tight junctions (9–15); different mechanisms of allergen recognition and uptake by DCs will be further described in the following sections.

## C-Type Lectin Receptors

C-type lectin receptors are mainly involved in the recognition of glyco-allergens. Diverse C-type lectin receptors (CLRs), such as dendritic cell-specific intracellular adhesion molecule 3-grabbing non-integrin (DC-SIGN) and mannose receptor (MR) on human DCs have been shown to be able to recognize and internalize allergens.

### Mannose receptor

This type-I integral transmembrane protein is primarily expressed by myeloid cells such as macrophages and DCs (4, 16). The extracellular portion of MR consists of three regions: a cysteine-rich domain, a fibronectin type II-like domain, and eight carbohydrate recognition domains (CRDs). Interestingly, DCs from patients with house dust mite (HDM) allergy have been shown to express higher levels of MR and to be more efficient in allergen uptake than DCs from non-atopic donors (17). More recently, it was reported that bronchoalveolar lavage fluid from patients with asthma and/or allergic rhinitis contains higher numbers of MR expressing myeloid-DCs compared to healthy controls (18). In terms of allergen uptake, *in vitro* studies have found that MR expressed on human monocyte-derived DCs is the main receptor for major allergens from HDM (*Der p* 1), dog (*Can f* 1), cockroach (*Bla g* 2), peanut (*Ara h* 1), and cat (*Fel d* 1) (19, 20). Similar studies have also highlighted MR’s role in allergen-induced Th2 cell differentiation where MR-deficient (MR^−^), as opposed to MR expressing (MR+), DCs failed to induce Th2 cell differentiation in response to Der p 1 in DC-T cell co-cultures from HDM atopic individuals. The bias toward Th1 cell polarization by MR^−^ DCs was shown to be partially mediated through the up-regulation of indoleamine 2,3-dioxygenase (IDO) activity in DCs (19). IDO is an enzyme that participates in tryptophan metabolism and is involved in many immune-regulatory processes in health and disease (21, 22). Further studies by the same group showed that MR recognition of major cat allergen *Fel d* 1 mediated the production of specific IgE and IgG1 antibodies in a mouse model of allergy (20). Other studies have shown that omega-1, a glycosylated T2 ribonuclease secreted by *Schistosoma mansoni* eggs, is recognized and internalized by DCs through MR and subsequently interferes with proteins synthesis and conditions DCs for Th2 priming (23). Collectively these data highlight MR’s role in allergen recognition and promotion of Th2-mediated immune responses.

### Dendritic cell-specific intracellular adhesion molecule 3-grabbing non-integrin

Dendritic cell-specific intracellular adhesion molecule 3-grabbing non-integrin is a type-II integral transmembrane protein that consists of four regions: a CRD, a hinge domain, and a transmembrane region connected to a cytoplasmic signaling domain (4, 24). DC-SIGN is mainly expressed by antigen presenting cells (APCs) and has been demonstrated to participate in the recognition of allergens from different sources, such as peanut, HDM, pollen, and dog (25–27). *In vitro* studies have been shown that DC-SIGN recognition and uptake of Der p 1 induces Th1 cell differentiation. On the contrary, DC-SIGN deficient DCs bias the response toward a Th2 profile (27). This is opposite to previous observations in MR^−^ DCs which seem to support Th1 differentiation (19). Interestingly, it has also been shown that Der p 1, using its enzymatic activity, can cleave (28) and induce the down-regulation (29) of cell surface DC-SIGN but not MR (28). In this context, we have previously proposed that the Th1/Th2 balance in response to allergen exposure can be determined by the cross-talk between these receptors and the level of their expression on DCs (4, 27). Accordingly, it is important to note that DCs from asthmatic patients show lower expression of DC-SIGN (29), which is in contrast to the high levels of MR expression reported in atopic individuals (17, 18).

### Toll-like receptors

The TLRs are type-I integral membrane receptors, each with an N-terminal ligand recognition domain constructed of tandem copies of a leucine-rich repeat motif, a single transmembrane helix, which participates in nucleic acid pathogen-associated molecular patterns (PAMPs) recognition, and a C-terminal cytoplasmic signaling domain known as Toll IL-1 receptor domain (30). There are more than 10 different TLRs identified in humans so far (31), with some of them being involved in allergen recognition or pathways that induces allergic responses. Within this context, mainly three different mechanisms have been proposed. Der p 2, a major allergen from HDM with lipid-binding activity, has been shown to induce signaling through TLR2 (32) and TLR4 (33) depending on the cell type involved. Due to its high homology with MD2, which participates in the recognition of lipopolysaccharide (LPS) by TLR4, Der p 2 forms a complex with TLR4 that signals similarly to MD2/TLR4 complex inducing innate immune activation (33). In addition, HDM extracts contaminated with flagellin can induce TLR5-dependent allergic responses in mice, however, the mechanism is still unclear (34). The second mechanism involves sensitization by nickel, which may not be relevant in the context of airway sensitization but highlights the importance of TLR4 on DCs in allergic reactions. Nickel and other bivalent metals such as cobalt induce a lipid-independent activation of TLR4, which is dependent on the presence of two histidine residues, promoting TLR4 dimerization and subsequent receptor activation independently of MD2 (35, 36). Finally, the last mechanism involves allergens such as Fel d 1 and Can f 6 that belong to lipocalin family and cause enhanced LPS-induced TLR4 activation in an MD2 dependent and independent manner respectively (37).

### High-affinity IgE receptor

FcεRI is a multimeric cell surface receptor that binds IgE with high-affinity. In humans, this receptor can be expressed by mast cells, basophils, eosinophils, platelets, monocytes, and DCs; however, in the last four cell types it adopts a trimeric (αγ2) structure instead of the classical tetrameric (αβγ2) structure (38–43). It has been previously suggested that cell-bound IgE participates in the presentation of aero-allergens by Langerhans cells (44). In addition, the presence of FcϵRI on Langerhans cells maximizes antigen uptake via specific IgE and subsequent presentation to T cells (45). In monocytes and peripheral blood DCs, FcϵRI has been shown to mediate IgE-dependent allergen presentation (41, 46). Further studies in DCs demonstrated that multimeric ligands of FcϵRI are delivered into a major histocompatibility complex class II compartment in a Cathepsin S-dependent pathway (47). *In vivo* experiments with a transgenic mouse model with human-like FcϵRI expression in DCs showed that after allergen capture DCs instructed naïve T cells to differentiate into allergen-specific Th2 cells at the site of allergen exposure (48). Taking into account the fact that higher expression of FcϵRI has been detected in atopic individuals (40, 42), this can lower the atopic individual’s threshold to mount allergen-specific T cell responses. All this highlights the fact that the high-affinity IgE receptor could play an important role in capturing allergens by DCs and subsequent presentation to T cell particularly in previously sensitized individuals with high levels of specific IgE; however, the precise role of IgE receptors and the role of other stimulatory and inhibitory Fc receptors (49, 50) in allergen presentation still need to be fully understood.

## Allergen Recognition by Epithelial Cells

**Figure 2:**
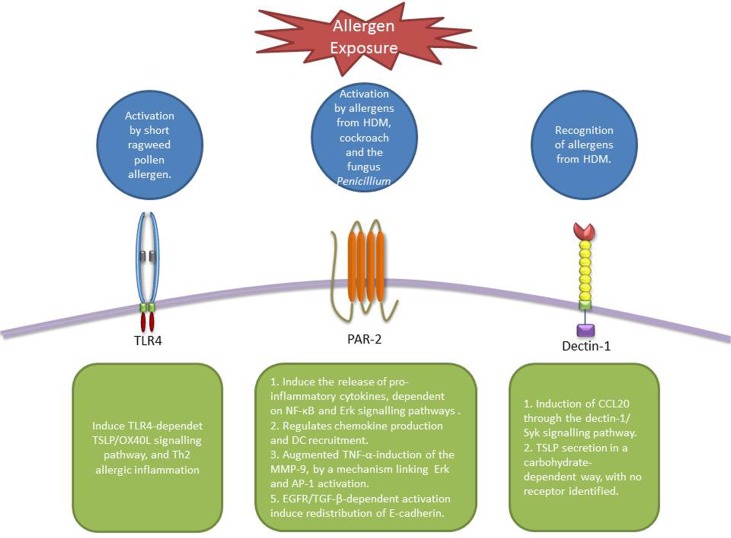
**Receptors involved in allergen recognition and uptake by AECs**. TLR4 can be activated by pollen allergens and activate TSLP-OX40L signaling pathway (88). PAR-2 is activated by different allergens from HDM, cockroach, and fungus inducing the production of cytokines and chemokines that modulate DC behavior (60, 61, 64–67). Dectin-1 is able to recognize allergens from HDM and induce the secretion of CCL20 (90). In addition, the secretion of TSLP induced by Der p 1 in AECs is thought to be carbohydrate-dependent, however, no receptor has been identified (92).

Airway epithelial cells constitute the first line of defense against pathogens and allergens by not only forming a physical barrier, but also through expressing a wide range of PRR, such as TLRs, CLRs, and protease activated receptor (PARs) (Figure 2). These receptors enable AECs to recognize microbial motifs and environmental allergens which lead to a cascade of events culminating in the release of cytokines, chemokine ligands, and danger signals which recruit and activate other immune cells.

### Protease activated receptors

Protease activated receptors are G-protein coupled receptors characterized by a self-activation mechanism following proteolytic cleavage of their extracellular N-terminal domain. There are four PAR members identified to date. PAR-1,-3,-4 respond to the protease thrombin, expressed primarily by cells in the vasculature; and are mainly involved in homeostasis and thrombosis. Conversely, PAR-2 is activated by trypsin-like serine proteases but not by trypsin and can be found in airways, vascular, skin, and intestinal cells, and mediates proliferative and inflammatory responses linked to tissue damage (51, 52). In addition, endothelial cells, epithelial cells, fibroblasts, and immune cells such as lymphocytes, monocytes, mast cells, neutrophils, eosinophils, macrophages, and DCs have been shown to express functional PAR-2 (53, 54). Due to its ability to respond to serine proteases, proteolytic allergens from diverse sources such as HDM, cockroach, pollen, or mold can act as exogenous activators of PAR-2 with implications in allergy and asthma.

Different cysteine and serine proteases from HDM, pollen, and the fungus *Penicillium* have been shown to increase epithelial permeability and disrupt the tight junctions by mainly targeting the transmembrane adhesion proteins occludin and zonula occludens-1 (9–15). In addition, it has been demonstrated that allergen-induced cytokine production, cell detachment and morphological changes in AECs is largely dependent on allergens’ protease activity (13, 55–58). Protease-dependant induction of IL-25 and thymic stromal lymphopoietin (TSLP) have been shown to be mediated by Erk and p38 MAPK pathways (58). More recently, it was demonstrated, in an *in vivo* model, that IL-33 also contributes to protease-dependent allergic airway inflammation (59).

Main allergens from HDM can activate PAR-2 and induce the release of pro-inflammatory cytokines (60, 61). However, there is some contradictory results showing that Der p 1-induction of IL-8 and IL-6 is independent of PAR-2 activation and dependent on nuclear factor κB (NF-κB) and Erk signaling pathways (62, 63). In the case of allergens from the German cockroach and the fungus *Penicillium*, this effect has been demonstrated to be mediated by the activation of Erk (64, 65). In an *in vivo* model of allergy, only when the allergens are administered through the mucosa, cockroach proteases regulate chemokine production and DC recruitment in a PAR-2-dependent way (66, 67). Furthermore, it was demonstrated that cockroach proteases augmented tumor necrosis factor (TNF)-α-induction of the matrix metalloproteinases-9, an enzyme that has been implicated in the pathogenesis of bronchial asthma (68, 69), by a mechanism linking PAR-2, Erk, and AP-1 activation (70). More recently, PAR-2-mediated allergic sensitization was shown to be associated with TNF signaling pathways (71).

In addition to the proteolytic activity of some allergens, one of the main soluble mediators that accounts for the increase in epithelial permeability is the vascular endothelial growth factor (VEGF). It has been shown that extract from cockroach increases bronchial airway epithelial permeability by inducing the release of VEGF (72). Besides, HDM extract can induce the secretion of VEGF by human pulmonary epithelial cells (73). Recently, it was demonstrated that HDM-induced redistribution of E-cadherin was mediated via epidermal growth factor receptor-dependent activation of PAR-2 and transforming growth factor-β (TGF-β) enhanced this signaling (74). Nevertheless, not only PAR-2 is directly involved in allergen-mediated cytokine production, it has also been shown that IL-8 production by AECs in response to allergens from the fungus *Penicillium* is dependent on PAR-1 and PAR-2 via activation of ERK1/2 (65). In addition, proteases from different fungal allergens induce the release of pro-inflammatory cytokines from human nasal polyp epithelial cells, leading to eosinophil and neutrophil migration, in a mechanism that could involve PAR-2 and PAR-3 (75).

### Toll-like receptor-4

As previously described, TLRs are widely expressed by both APCs and epithelial cells and recognize conserved microbial structures and as such play a key role in controlling adaptive immune responses. LPS is recognized by TLR4 with the participation of the accessory proteins including CD14, LPS binding protein, and MD2. This leads to the recruitment of the signaling adapter protein MyD88, the activation of the transcription NF-κB among others, and the expression of pro-inflammatory cytokines (76). TLR4 (77–80), MyD88 (79, 81, 82), and NF-κB (83–86) have been shown to be crucial in the elicitation of allergic Th2 immune responses. Different studies using knock-out mice have demonstrated the importance of TLR4 expression in both hematopoietic radiosensitive and structural radioresistant cells in the induction of TLR4-dependent Th2 responses to intranasal allergens in the presence of endotoxin (77–80). In addition, it has been shown that the LPS dosage is crucial in driving either Th1 or Th2 responses, with lower levels of LPS inducing Th2 responses to inhaled allergens in a mouse model of allergic sensitization (87). Recently, it was shown that short ragweed pollen acts as a TLR4 agonist, initiating TLR4-dependet TSLP/OX40L signaling pathway, triggering Th2 allergic inflammation (88).

### C-type lectin receptors

C-Type Lectin Receptors are receptors that recognize oligosaccharide moieties among other molecular patterns on antigens including allergens (89). CLR’s role in allergen recognition and uptake by DCs is well established (4); moreover, they have also been shown to participate in allergen recognition by AECs. It was demonstrated that HDM induction of CCL20 by AECs was not protease or TLR4/2 dependant; however, it was mediated by β-glycan moieties within HDM extract. This effect was specific for HDM because other allergens, such as cockroach and ragweed, failed to induce this response (90). The authors suggest the involvement of the dectin-1/Syk signaling pathway, since Syk inhibition abrogated the HDM-induced CCL20 production (91). More recently, our group demonstrated that TSLP secretion by AECs upon stimulation with Der p 1 was at least partly carbohydrate-dependent. In addition, DC uptake of deglycosylated Der p 1 was considerably decreased compared with its natural (glycosylated) counterpart, indicating that glycosylation of allergens plays a crucial role in their recognition by immune and non-immune cells (92).

## Cross-Talk between Dendritic and Epithelial Cells

**Figure 3:**
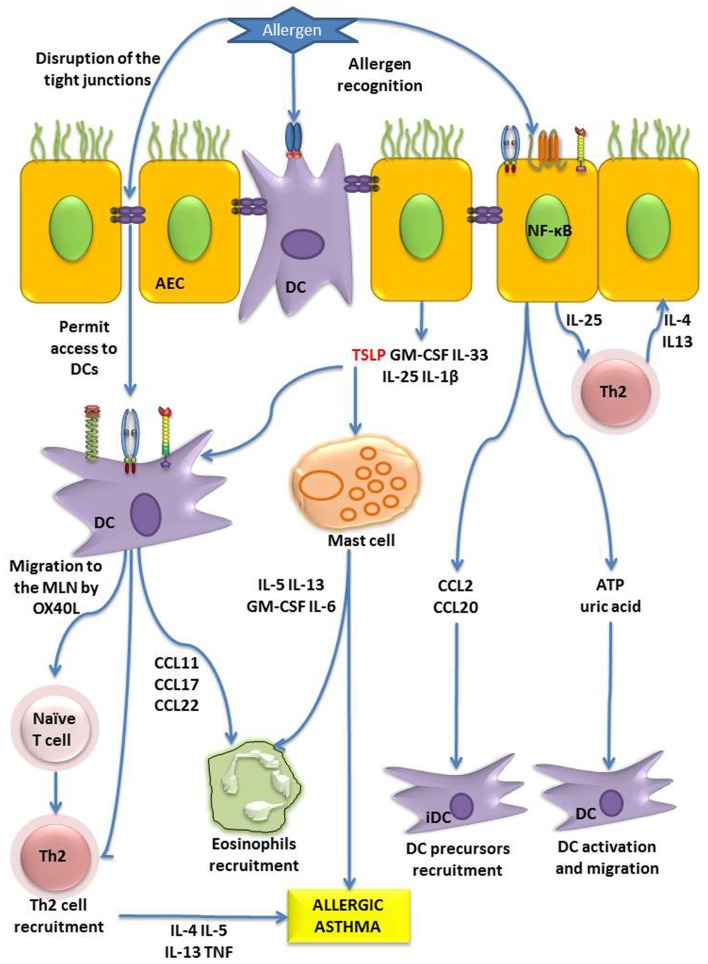
**Role of AECs in allergen sensitization**. AECs can directly recognize allergens by PARs, TLRs or CLRs, and through NF-κB signaling, they induce the production of the cytokines (TSLP, GM-CSF, IL-33, IL-25, IL-1β), chemokine ligands (CCL2, CCL20), and danger signals (adenosine triphosphate, uric acid). TSLP has diverse effects on DCs and mast cells, which in absence of the cytokine IL-12 can lead to recruitment of Th2 cells and (eosinophils, Th2 polarization, and the onset of allergy or allergic asthma. Moreover, IL-25 can activate Th2 cells to produce pro-allergenic cytokines. On the other hand, DCs are able to form tight junction with AECs, and allergen can digest the tight junction and both phenomena contribute to facilitate the allergen sensitization process (6, 7).

Due to their strategic location at the interface of external-internal environments, AECs are able to modulate and coordinate immune responses. Ample data have shown that the cross-talk between DCs and AECs is crucial in driving allergen-induced Th2 responses (6, 7). As previously described, the ligation of different PRR on AECs results in the secretion of chemokines (93–95), that attract DCs, and cytokines that induce DC maturation and activation. However, there are some contradictory results indicating that inducible signals driven by LPS in non-hematopoietic tissues such as AECs do not play an essential role in DCs activation (96). On the other hand other studies have demonstrated that AECs can induce DC maturation after LPS inhalation (81). Nevertheless a range of cytokines including TSLP, IL-33, IL-25, IL-1β, and granulocyte macrophage colony-stimulating factor (GM-CSF) are known to be secreted by AECs after allergen challenge (6, 7) which are able to modulate DCs function (Figure 3). Here we focus on TSLP as a key cytokine in initiation and maintenance of allergic responses.

## Role of TSLP

Thymic stromal lymphopoietin is a 140-amino acid four-helix-bundle cytokine that belongs to the IL-2 family of cytokines. This cytokine is mainly produced by AECs and is able to modulate DC by binding to its receptor complex composed by the TSLP receptor (TSLPR) and the IL-7 receptor (IL-7R) (97). This induces the production of Th2-attracting chemokines such as CCL22 and CCL17, and primes naïve T cells to produce IL-5, IL-4, TNF-α, IL-13, whereas down-regulate IL-10 and IFN-γ (98, 99) even in the absence of DCs in an IL-4 dependant way (100). TSLP induces the expression of OX40L in DCs which in turn has been shown to trigger Th2 cell polarization in the absence of IL-12 (101). Conversely, TSLP is also able to induce IL-12 secretion after CD40 ligation in DCs however still maintaining its Th2-polarizing effect (102). Furthermore, DCs activated with TSLP were able to induce the expansion of Th2 memory cells and help to maintain their phenotype (103).

Human epithelial cells can produce TSLP in response to diverse stimuli, such as microbial products, physical injury, ambient particulate matter, protease allergens, and either pro-inflammatory or Th2-polarizing cytokines (58, 104–108). Protease allergens induce TSLP in a PAR-2-dependent way (109) with the involving of MAPK signaling pathway (58) however inflammatory cytokines induction of TSLP is NF-κB signaling dependent (104, 106). In addition to DCs, TSLP can also activate mast cells and CD34+ blood hematopoietic progenitor cells to produce Th2 cytokines and in that way induce the innate phase of allergic immune responses (105, 110). Finally, TSLP can interfere with regulatory T cell development impairing the balance between tolerance and inflammation (111). Keratinocytes too can secrete functional TSLP after stimulation with pro-inflammatory or Th2-driven cytokines, and induces DC activation in human skin lesions of atopic dermatitis (112). Recently, it was demonstrated that DCs can also produce TSLP in response to TLR stimulation. Moreover, interestingly DCs from mice challenged with HDM extract express higher mRNA levels of TSLP than epithelial cells (113).

*In vivo* experiments have shown that TSLPR knock-out mice exhibited strong Th1 responses while Th2 responses were impaired and they failed to develop an inflammatory response to allergen challenge to lung (114) or skin (115). In addition, skin and lung over-expression of TSLP induces atopic dermatitis (116) and airway inflammation respectively (117). In an experimental model of allergic conjunctivitis it was demonstrated that after topical allergen challenge mucosal epithelial cells produce high levels of TSLP compared with controls leading to induction of allergic inflammation through the TSLP-OX40L signaling pathway (118). More recently, it was shown that the soluble TSLPS antagonist, comprised of the extracellular domain of the murine TSLPR and an IgG2a Fc tail, reduced the severity of airway inflammation by regulating DC function (119).

Furthermore, it has been demonstrated that TSLP and Th2-attracting chemokines are increased in airways of asthmatic subjects compared with normal controls (120). In addition, different studies have shown an association between genetic polymorphisms in the human IL-7Rα chain and TSLP genes with allergy, allergic rhinitis, and bronchial asthma further highlighting a possible link between these proteins and allergy (121–123).

## Conclusion

Dendritic cells are professional APCs and sentinels of the immune system that efficiently sample allergens in the airways leading to a cascade of events that culminates in the induction of Th2 type immune responses. DCs are able to recognize and internalize allergens from diverse sources through expression of a plethora of receptors such as CLRs, TLRs, and FcϵR. Recently, the role of AECs as key players in the modulation and induction of DCs in the airways has been highlighted. Like DCs, AECs are able to recognize allergens through several PRRs including PARs, CLRs, and TLRs. This further leads to the production of different cytokines, chemokines, and danger signals with the ability to initiate and propagate immune responses to allergens. Allergens have diverse molecular features such as specific oligosaccharide moieties, protease activity, lipid-binding properties, among others that can facilitate their recognition by immune and non-immune cells and contributes to their “allergenicity.” Better understanding of the molecular basis of early events at the interface of allergens and their receptors and the key soluble mediators/signaling pathways involved could lead to development of more effective therapeutic strategies for allergic diseases including allergic asthma. For instance, due to the contribution of TLRs and CLRs in the recognition of allergen by both DCs and AECs, agonist and antagonists to those receptors may provide new therapeutic targets to modulate allergic responses. In addition, different studies have highlighted the role of sugar moieties on allergens in their recognition and internalization by immune cells. Accordingly, different “glycoforms” of allergens with immunoregulatory properties could be developed and used in allergen-specific immunotherapy strategies. Finally, diverse intracellular and extracellular molecules have been implicated in the process of allergen recognition and sensitization. Further studies to decipher these mechanisms could pave the way for the rational design of more effective therapeutic entities for the treatment of allergic diseases.

## Conflict of Interest Statement

The authors declare that the research was conducted in the absence of any commercial or financial relationships that could be construed as a potential conflict of interest.

## Abbreviations

AEC, airway epithelial cell; APC, antigen presenting cell; CLR, C-type lectin receptor; CRD, carbohydrate recognition domain; DC, dendritic cell; DC-SIGN, dendritic cell-specific intracellular adhesion molecule 3-grabbing non-integrin; FcϵR, Fcϵ receptor; GM-CSF, granulocyte macrophage colony-stimulating factor; HDM, house dust mite; IDO, indoleamine 2,3 dioxygenase; LPS, lipopolysaccharide; MR, mannose receptor; NF-κB, nuclear factor κB; PAMP, pathogen-associated molecular pattern; PAR, protease activated receptor, PRR, pattern recognition receptor; TGF-β, transforming growth factor-β; TLR, toll-like receptor; TNF, tumor necrosis factor; TSLP, thymic stromal lymphopoietin; VEGF, vascular endothelial growth factor.
